# Cross-field strength and multi-vendor reliability of MagDensity for MRI-based quantitative breast density analysis

**DOI:** 10.1371/journal.pone.0316076

**Published:** 2025-06-24

**Authors:** Jia Ying, Renee Cattell, Chuan Huang

**Affiliations:** 1 Department of Radiology and Imaging Sciences, Emory University School of Medicine, Atlanta, Georgia, United States of America; 2 Department of Biomedical Engineering, Stony Brook University, Stony Brook, New York, United States of America; 3 Department of Radiation Oncology, Renaissance School of Medicine at Stony Brook University, Stony Brook, New York, United States of America; 4 Department of Biomedical Engineering, Georgia Institute of Technology and Emory University, Atlanta, Georgia, United States of America; Linköping University, SWEDEN

## Abstract

**Purpose:**

Breast density (BD) is a significant risk factor for breast cancer, yet current assessment methods lack automation, quantification, and cross-platform consistency. This study aims to evaluate the reliability and cross-platform consistency of MagDensity, a novel magnetic resonance imaging (MRI)-based quantitative BD measure, across different imaging platforms.

**Methods:**

Ten healthy volunteers participated in this prospective study, undergoing fat-water MRI scans on three scanners: 3T Siemens Prisma, 3T Siemens Biograph mMR, and 1.5T GE Signa. Great effort was made to schedule all scans within a narrow three-hour window on the same day to minimize any potential intra- or inter-day variations, requiring substantial logistical coordination. BD was assessed using the MagDensity technique, which included combining magnitude and phase images, applying a fat-water separation technique, employing an automated whole-breast segmentation algorithm, and quantifying the volumetric water fraction. Agreement between measures across scanners was analyzed using mean differences, two-tailed t-tests, Pearson’s correlation, and Bland-Altman analysis.

**Results:**

MagDensity measures obtained from the two 3T Siemens scanners demonstrated no statistically significant differences, with high correlation (Pearson’s r > 0.99) and negligible mean differences (< 0.2%). Cross-platform comparison between the 3T Siemens and the 1.5T GE scanners showed larger mean differences (< 4.2%). However, after applying linear calibration, these variations were reduced to within ±0.2%, with strong inter-scanner correlation maintained (Pearson’s r > 0.97).

**Conclusion:**

MagDensity showed strong intra-vendor consistency and promising cross-platform reliability after leave-one-out calibration. While full standardization remains a long-term goal, these findings provide clear evidence that scanner-related variability can be effectively mitigated through calibration. This technique offers a step further toward more consistent MRI-based BD quantification and may help enable broader clinical implementation.

## Introduction

Breast cancer remains a significant global health challenge, being a leading cause of morbidity and mortality among women. Extensive research has identified various risk factors, with higher breast density (BD) recognized as one of the most crucial, closely linked to an increased risk of developing breast cancer [1,2]. BD has been incorporated into several breast cancer risk models for personalized risk assessment, such as Tyrer-Cuzick model, resulting in improved risk discrimination [3–7]. Its critical role was further highlighted by legislative reforms initiated in 2011, when more than half of the U.S. states passed laws requiring healthcare providers to inform patients with dense breasts [8]. This significant move acknowledges that higher BD increases cancer risk and may necessitate supplemental screening measures [9]. Moreover, longitudinal changes in BD have been examined in clinical trials as surrogate measures for evaluating the efficacy of hormone therapies (e.g., tamoxifen and aromatase inhibitors) used for the prevention and treatment of breast cancer [10,11]. A previous study demonstrated that a reduction in mammographic density after 12–18 months of tamoxifen therapy was associated with a reduction in cancer risk [12]. Thus, a sensitive BD measurement is useful not only for evaluating the risk of developing breast cancer, but also for monitoring the effectiveness of preventive interventions and tailoring treatment strategies for individual patients.

The current standard of care for assessing BD includes mammography, followed by a qualitative categorization into one of the four major categories according to the Breast Imaging Reporting and Data System (BIRADS): almost entirely fatty, scattered areas of fibroglandular tissue, heterogeneously dense, and extremely dense breasts. However, this qualitative approach has inherent subjectivity, introducing potential variability in clinical interpretation [13,14]. Additionally, the reliability of BD measurements using mammography is compromised by two-dimensional (2D) nature of projection imaging. Although advanced mammographic techniques, such as spectral mammography and digital breast tomosynthesis, have provided improved tissue characterization and density quantification [15,16], they continue to rely on ionizing radiation and breast compression and do not provide fully isotropic three-dimensional (3D) volumetric data. These constraints may limit their effectiveness for frequent monitoring and precise longitudinal assessment of BD changes.

Due to these limitations, breast magnetic resonance imaging (MRI) has emerged as a promising contender for these challenges due to its distinct advantages including offering true 3D imaging without exposure to ionizing radiation or breast compression. Previously, Ding et. al. [17] proposed a quantitative MRI-based BD measure known as MagDensity, which utilizes the Dixon fat-water decomposition method, combined with whole-breast segmentation. This technique demonstrated high test–retest reproducibility (i.e., intra-scanner) and improved quantitative accuracy by correcting signal biases typically seen in MRI fat-water decomposition imaging—specifically, biases arising from differences in proton density, T1 relaxation effects, and residual water signal in adipose tissue [18]. MagDensity measure has also been successfully used as an outcome measure in several clinical trials (clinical trial numbers: NCT01761877 and NCT02028221) [19,20] to longitudinally monitor changes in BD.

While MRI-based methods have established benefits, their consistency and reproducibility across different scanner models, field strengths, and vendors have not been extensively investigated. Reproducibility is a fundamental aspect of scientific research, strongly advocated by the National Institutes of Health as a means to ensure that biomedical research findings can be reliably utilized in clinical settings. For women, particularly those at an elevated risk of breast cancer, who may need multiple MRI scans at different healthcare facilities over their lifetime, the cross-platform consistency of BD measurements across different scanner models, field strengths, and vendors becomes paramount for clinical application [21]. Ensuring that BD measures are generalizable across diverse MRI systems is critical for integrating these assessments into standard clinical workflows, thereby facilitating accurate and consistent breast cancer risk evaluation and monitoring across varied medical environments. Therefore, a dedicated cross-scanner reliability study is critical to ensure the robustness of BD measurements across different platforms. However, scheduling scans for different scanners on the same day poses significant challenges due to the need for precise coordination and availability of multiple MRI systems within a narrow timeframe. This requirement is crucial for a successful cross-scanner study because factors such as menstrual cycle phases and weight fluctuations can affect BD measurements, thereby undermining the reliability of the analysis [22].

In this study, we aimed to evaluate the reliability of MagDensity for BD quantification using data acquired within a narrow three-hour timeframe (for each participant) from three scanners with different field strengths (3T and 1.5T) and vendors (Siemens and GE). To our knowledge, this work is among the first few studies to explicitly evaluate the cross-scanner reliability for a quantitative MRI-based BD measurement.

## Materials and methods

### Study population

This prospective study was approved by the Stony Brook Institutional Review Board, and written informed consent was obtained from all participants. The recruitment period began on August 1, 2019 and concluded on November 8, 2019. All assessments and analyses were performed in accordance with relevant guidelines and regulations. Inclusion criteria of the study were: i) females within the age range of 18–75 years; ii) no prior history of breast cancer or known breast-related diseases, iii) no contraindications to MRI, and iv) ability to provide informed consent. Exclusion criteria included: i) long-term use of non-steroidal anti-inflammatory drugs (NSAIDs), ii) claustrophobia or an elevated risk of cardiac arrest, iii) unable to lie comfortably on scanner bed for 20 minutes, iv) contraindication to MRI, and iv) cognitively impaired or not able to provide informed consent.

### MRI protocol

Each participant underwent three fat-water breast MRI scans using 3T scanners (Siemens Prisma and Siemens Biograph mMR) and a 1.5T scanner (GE Signa). All participants were scanned in the prone position, identical to current practice for breast MRI. Data for each participant were acquired within a three-hour window to mitigate the impact of physiological variations, such as menstrual cycle-related fluctuations and weight changes. The scanning parameters for each scanner are summarized in Table 1. Note that the variations in scanning parameters were intentionally introduced as part of the study’s methodology to assess the reliability of our BD assessment technique. The sequences and parameters were chosen based on existing clinical sequences.

**Table 1:** Scanning parameters used for acquisition.

Scanner Parameter	3T Siemens Prisma	3T Siemens Biograph mMR	1.5T GE Signa HDxt
**Coil**	16-channel breast (Sentinelle, Erlangen, Germany)	8-channel breast (Sentinelle, Erlangen, Germany)	Sentinel 4-channel biopsy table (Sentinelle, Erlangen, Germany)
**Orientation**	Axial	Axial	Axial
**Sequence**	3D Cartesian six-echo GRE	3D Cartesian six-echo GRE	EFGRE3D six-echo
**Acquisition matrix**	256 × 152	256 × 152	512 × 372
**Pixel size**	1.97 × 1.97 mm^2^	1.97 × 1.97 mm^2^	0.625 × 0.625 mm^2^
**Flip angle**	6 degrees	6 degrees	12 degrees
**Slice thickness/gapping**	4/0 mm	4/0 mm	2/0 mm
**Phase encoding direction**	A – P	A – P	A – P
**Number of averages**	1	1	1
**Repetition time**	21.0 ms	21.0 ms	22.4 ms
**Echo time**	1.37, 2.66, 4.92, 6.15, 7.38, 8.81 ms	1.37, 2.66, 4.92, 6.15, 7.38, 8.81 ms	2.88, 6.04, 9.20, 12.35, 15.51, 18.66 ms

GRE: gradient echo; EFGRE3D: enhanced fast gradient echo three-dimensional.

* Note that the variations in scanning parameters were intentionally introduced as part of the study’s methodology to assess the reliability of our BD assessment technique.

### Image processing

The magnitude and phase images extracted from the scanner were combined to generate complex images. A fat-water separation technique called iterative decomposition of water and fat with echo asymmetric and least-squares estimation (IDEAL) [23] was used to reconstruct fat-only, water-only, and fat fraction imaged (representing the relative percentage amount of fat signals in each voxel) from multi-echo complex MRI data. Phase correction was applied to account for both linear and constant phase components across the echo dimension. The image processing steps were performed using in-house software developed in Matlab R2020b (MathWorks, Natick, MA).

### Whole-breast segmentation

We employed an established automated whole-breast segmentation algorithm [24] to delineate breast regions. The algorithm included finding the most similar breast templates from a previously built dictionary, followed by an image registration step to register the chosen templates to the acquired breast data, while concurrently applying the transformations to the associated template masks to generate the masks. Introducing image registration in the segmentation process represents a significant advancement over previous methods, providing a more reliable foundation for subsequent analyses [24]. For an in-depth explanation, please refer to ref [24] for details.

### MRI-based BD measure – MagDensity

MagDensity measure is an MRI-based metric previously introduced by Ding et. al [17] to quantify BD through volumetric assessment of fibroglandular tissue. This novel BD measurement takes into consideration both the volume and spatial distribution of fibroglandular tissue within the breast. The cornerstone of this measurement is the voxel-wise calculation of the volumetric water fraction, referred to as “FraWater”, which corrects for signal bias in fat fraction maps. This bias arises from proton density and T1 differences between fat and water, as well as the presence of residual water signal in adipose tissue. To perform this correction, a linear model is employed at each pixel:


$$\left\{ \begin{array}{c} S_{fat}=aV_{fat}+bV_{water}\\ S_{water}=cV_{fat}+dV_{water} \end{array}\right.\;$$ (1)

Here, *S*_*fat*_ and *S*_*water*_ denote the signal intensities of fat and water, respectively; *V*_*fat*_ and *V*_*water*_ are the corresponding fat and water volumes, respectively; and *a*, *b*, *c*, *d* are the correction factors. These correction factors were estimated individually for each subject by sampling signal intensities from reference regions of “pure” fat (i.e., subcutaneous fat within the breast; *V*_*fat*_ = 1, and *V*_*water*_ = 0) and “pure” water (i.e., pectoral muscle posterior to the breast; *V*_*fat*_ = 0, and *V*_*water*_ = 1) on the fat fraction map. In this study, the correction factors were first determined individually for each image and subsequently averaged to derive a scanner-specific set. Using these correction factors, a bias-corrected volumetric water fraction map—FraWater—can be computed across the entire breast. MagDensity is then defined as the mean FraWater within the segmented breast volume. This correction improves the accuracy of water signal quantification and, by extension, the reliability of BD assessment. Further technical details are described previously [17].

### Statistical analysis

Using MagDensity [17] in combination with the improved breast segmentation method [24], a robust and reproducible framework for MRI-based BD quantification was established. MagDensity measures were calculated separately for the left and right breasts of each subject across all three scanners.

Agreement between the measures across the scanners was assessed using mean differences, two-tailed t-tests, Pearson’s correlation, and Bland-Altman analysis. The significance level was set at 0.05. All analyses were conducted using MATLAB R2020b (MathWorks, Natick, MA).

## Results

**Fig 1:**
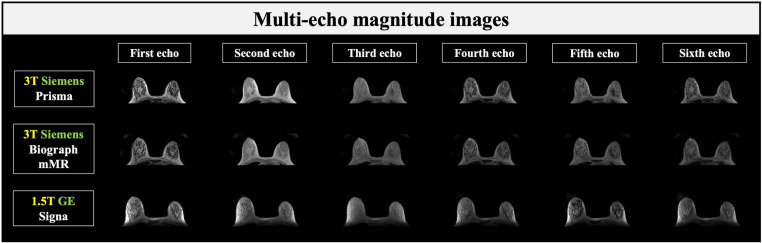
Multi-echo magnitude images from different scanners. Multi-echo magnitude images acquired from a representative participant, using 3T Siemens Prisma (top row), 3T Siemens Biograph mMR (middle row), and 1.5T GE Signa (bottom row).

**Fig 2:**
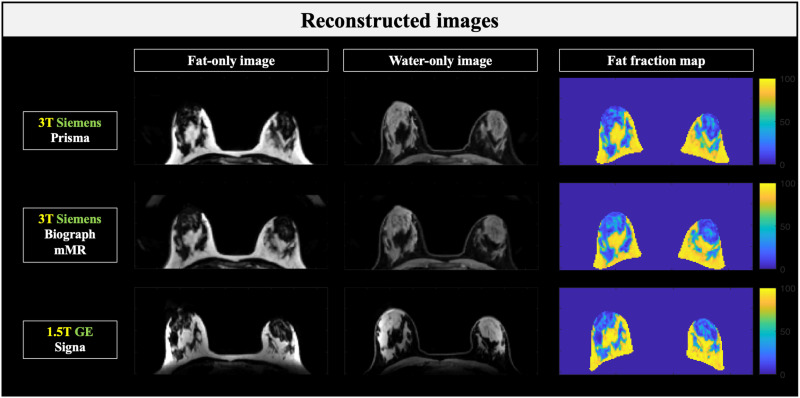
Reconstructed images using the multi-echo data. Reconstructed images from a representative participant, including fat-only images (left column), water-only images (middle column), and regions of interest on the fat fraction maps (right column) used for MagDensity calculations.

Ten healthy female volunteers aged 19–29 years (22.7 ± 3.3), with no known breast disease, were enrolled in the study. A total of 20 breast datasets (left and right breasts) were obtained. Fig 1 shows the multi-echo images from the three scanners for a representative volunteer. Fig 2 displays the reconstructed fat-only and water-only images derived from the echo data, along with the corresponding fat fraction maps used for MagDensity calculation.

**Fig 3:**
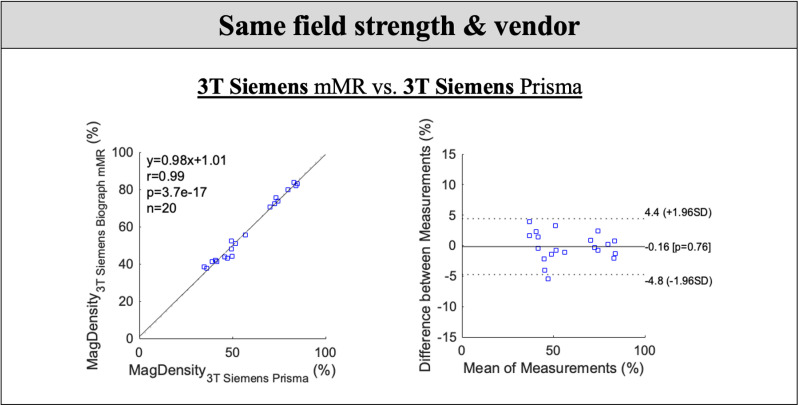
Pearson’s correlation and Bland–Altman analyses between the MagDensity measures of 3T Siemens Prisma and 3T Siemens Biograph mMR. Pairwise t-test showed no statistically significant difference between the MagDensity measures (p > 0.05) with a Pearson coefficient larger than 0.99 (p < 0.001). The Bland-Altman analysis showed a mean bias of −0.16% with 95% limits of agreement between −4.8% and 4.4%.

For MagDensity measures from the same field strength (3T) and the same vendor (Siemens), the observed cross-scanner mean differences were small (within 0.2%). Pairwise t-test found no statistically significant difference between the MagDensity measures of 3T Siemens Prisma and 3T Siemens Biograph mMR (*p* > 0.05). Bland-Altman analysis showed a mean bias of –0.16% with 95% limits of agreement ranging from –4.8% to 4.4%. The results are summarized in Table 2, and Fig 3 presents the Pearson’s correlation and Bland–Altman plots.

**Table 2:** Comparison of MagDensity measures between the same field strength (3T) and the same vendor (Siemens).

	3T Biograph mMR vs. 3T Prisma
**Mean Δ**_**1–2**_	−0.163%
***p* (t-test)**	0.760
**Pearson correlation coefficient (r)**	0.991

Δ_1–2_: signed difference between the former and latter scanners.

**Fig 4:**
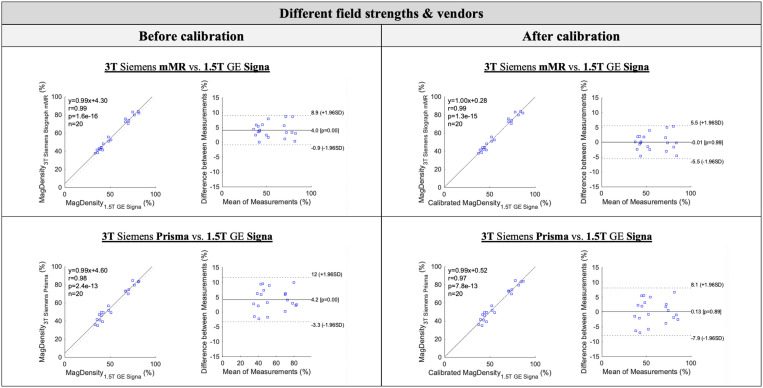
Pearson’s correlation and Bland-Altman analyses between the MagDensity measures of 3T Siemens Biograph mMR/Prisma vs. 1.5T GE Signa (before and after calibration). After calibration, pairwise t-test showed no statistically significant difference between the MagDensity measures between 3T Siemens Biograph mMR/Prisma and 1.5T GE Signa (p > 0.05) with Pearson coefficients larger than 0.97 (p < 0.001). The calibration was performed in a leave-one-out manner. For measures between 3T Siemens Biograph mMR and 1.5T GE Signa, the Bland-Altman analysis showed a mean bias of −0.01% with 95% limits of agreement between −5.5% and 5.5%. For measures between 3T Siemens Prisma and 1.5T GE Signa, the Bland-Altman analysis showed a mean bias of 0.13% with 95% limits of agreement between −7.9% and 8.1%.

For MagDensity measures across different field strengths and vendors (3T Siemens vs. 1.5T GE) as well as with different types of sequences, although they remained highly correlated (r > 0.97), slightly larger mean differences were observed, with a mean difference of 4.02% (Biograph mMR vs. GE Signa) and 4.19% (Prisma vs. GE Signa). However, this bias can be easily corrected by a linear calibration between the MagDensity measures of 3T Siemens Biograph mMR vs. 1.5T GE Signa (no statistically significant difference between the two Siemens scanners). Bland-Altman analysis showed a mean bias of 4.0% with 95% limits of agreement from –0.9% to 8.9% for the comparison between 3T Siemens Biograph mMR and 1.5T GE Signa. For 3T Siemens Prisma versus 1.5T GE Signa, the mean bias was 4.2%, with 95% limits of agreement ranging from –3.3% to 12%. After the calibration, these biases were largely corrected: the mean bias was reduced to –0.01% (limits: –5.5% to 5.5%) for Biograph mMR vs. Signa and 0.13% (limits: –7.9% to 8.1%) for Prisma vs. Signa. All calibrated 1.5T GE Signa MagDensity values were obtained using leave-one-out cross-validation. The observed cross-field/vendor mean differences were within ±0.2%. Pairwise analysis showed no statistically significant difference between the MagDensity measures between 3T Siemens Prisma/Biograph mMR and 1.5T GE Signa (*p* > 0.05). The results are summarized in Table 3, and Fig 4 shows the Pearson’s correlation and Bland–Altman analyses.

**Table 3:** Comparison of MagDensity measures across different field strengths and vendors (3T Siemens vs. 1.5T GE) before and after calibration.

	3T Biograph mMR vs. 1.5T Signa		3T Prisma vs. 1.5T Signa	
	Before calibration	After calibration	Before calibration	After calibration
**Mean Δ**_**1–2**_	4.023%	−0.007%	4.186%	0.129%
***p* (t-test)**	<0.001	0.991	<0.001	0.889
**Pearson correlation coefficient (r)**	0.989	0.986	0.976	0.972

_1-2_: signed difference between the former and latter scanners.

* Note that the calibration was performed in a leave-one-out manner.

## Discussion

BD remains is a well-recognized biomarker for breast cancer risk and is routinely incorporated into both risk prediction models and clinical trial endpoints. In this study, we evaluated the cross-scanner reliability of MagDensity, a quantitative MRI-based BD measure, using data from different vendors (Siemens and GE) and field strengths (3T and 1.5T). Our findings demonstrate that MagDensity yields consistent measurements across varying imaging platforms, with high correlation and minimal post-calibration bias.

When comparing MagDensity measures from scanners of the same vendor, sequence, and field strength (i.e., 3T Siemens), negligible differences were observed. The strong correlation (r = 0.99, *p* < 0.001) suggests consistent performance within the same technological environment and high intra-vendor reliability, affirming findings from previous test-retest studies [16]. Expanding the analysis to include different field strengths, vendors, and sequences (i.e., 3T Siemens vs. 1.5T GE) unveiled a small bias of approximately 4%, suggesting small initial inter-vendor, -field strength, and -sequence variability. Several factors may have contributed to this cross-platform difference. Most notably, differences in field strength (3T vs. 1.5T) may influence fat–water quantification. While higher field strength systems provide greater chemical shift dispersion and potentially higher signal-to-noise ratio, they are also more susceptible to B₀ field inhomogeneities and faster T2* decay, which may introduce variability in measurements, particularly in regions with complex tissue composition. The flip angle (12° vs. 6°), slice thickness (2 mm vs. 4 mm), repetition time (22.4 ms vs. 21.0 ms), and particularly echo times (ranging from 2.88 to 18.66 ms vs. 1.37 to 8.81 ms) also varied substantially between systems. These factors may influence GRE-based Dixon reconstructions by affecting fat–water signal modeling and T2 decay behavior. However, the exact contributing factor(s) of this bias, although scientifically interesting, is beyond the scope of this work. Also, this represents a “worst-case” scenario, as the images used for MagDensity calculation were acquired on different scanners, at different field strengths, and with different acquisition sequences and parameters. Importantly, this bias can be effectively addressed through linear calibration between the measures of the 3T Siemens Biograph mMR and the 1.5T GE Signa, leading to aligned values within a margin of ±0.2%. Although this calibration was performed based on same-subject imaging and is not directly translatable to routine clinical workflows, it provides proof-of-concept evidence that scanner-specific variation can be accounted for when appropriate reference data are available. It is worth noting that our primary objective was not to assert the absolute uniformity or direct equivalence of BD measurements across diverse scanners and configurations. Rather, our primary aimed to demonstrate that inter-scanner differences—when present—can be addressed through calibration, thereby improving measurement consistency across platforms. In essence, while we acknowledge the inherent intricacies arising from alterations in field strength, scanning protocols, and scanner vendors, our study underscores the feasibility of mitigating these complexities through a calibration approach. This methodology ensures that BD measurements obtained across different platforms maintain a consistent level of reliability, even in scenarios where multiple variables undergo simultaneous adjustments during the scanning process.

Another potential approach for calibration could be to develop a calibration phantom. By scanning the phantom on different scanners, a calibration algorithm could be built. While this approach offers potential, it presents significant logistical and practical challenges, such as the need for specialized fabrication, rigorous validation to ensure long-term accuracy and stability, and ongoing maintenance. Considering these substantial requirements and the early-stage nature of MagDensity’s implementation, this strategy was deemed outside the scope of our current study. Nonetheless, as MRI-derived breast density assessment advances towards broader clinical adoption, incorporating calibration phantoms into routine workflows could become a valuable future enhancement for further refining cross-platform consistency.

The exploration of BD as a predictive marker for breast cancer risk has drawn considerable attention, with mammographic density assessment via BI-RADS remaining the clinical standard. However, its inherent subjectivity and reliance on 2D projections have prompted the development of more objective alternatives [13,14,25]. Even with the integration of advanced techniques, these methods remain fundamentally projection-based, involve ionizing radiation, and are not suitable for extremely dense breasts [15,16,26–29]. MRI-based techniques offer a promising alternative [30–34]. Among these, Dixon-based MRI methods have gained attention for their ability to separate fat and water components with high precision, enabling more direct quantification of fibroglandular tissue. Petridou et al. [32] applied a two-point Dixon technique in conjunction with multi-atlas segmentation on a wide-bore 3T MRI scanner (Philips Healthcare) to estimate breast fat volume, reporting high correlation with BI-RADS scores and consistency between pre- and post-contrast acquisitions. Wengert et al. [33,34] developed a fully automated, user-independent pipeline using 3D Dixon imaging, and intensity histogram classification, demonstrating strong intra-scanner reproducibility in a single-vendor environment. However, neither study addressed potential variability introduced by differences in scanner model, field strength, or acquisition protocol. These factors are critical when considering the integration of MRI-based BD assessment into multicenter studies or longitudinal clinical applications.

To address this gap, we assessed the cross-platform consistency of the MagDensity, using same-subject data acquired across different scanner models, vendors, and field strengths. This study builds upon the previously established MagDensity framework [17] and introduces several important advancements. All scans were conducted within a tightly controlled three-hour window to minimize physiological variability, a design that is logistically challenging but essential for ensuring biological comparability. We also employed an improved breast segmentation algorithm [24], previously demonstrated to outperform the original implementation used in the initial MagDensity publication. Notably, we found that MagDensity values showed good agreement across platforms even before calibration. To support generalizability beyond matched same-subject acquisitions, we applied a leave-one-out cross-validation approach to derive scanner-specific calibration models, allowing the method to be transferable across different imaging systems without requiring repeated imaging of the same individual. This cross-platform evaluation is crucial for ensuring the reliability and accuracy of BD measurements over time. A simulation based on a sample size of 10 subjects demonstrated that reducing the standard deviation between two measures from 1.42% to 1.11% enabled detection of a true 1% change in BD with statistical significance (p < 0.05), highlighting the importance of measurement precision in capturing subtle longitudinal changes [24]. Such sensitivity is particularly important in monitoring high-risk women or evaluating the impact of interventions, especially in postmenopausal women where changes are expected to be small in response to endocrine therapies [19]. Moreover, consistent cross-platform measurements enable pooling data from different studies or centers, increasing the statistical power and generalizability of findings related to BD changes. Last but not least, since BD can be influenced by various hormonal and physiological factors, including menopause, pregnancy, and lactation, robust longitudinal tracking can provide insights into the effects of these life stages on BD, thereby contributing to a more comprehensive understanding of individual breast health dynamics [35,36].

There are several comments worth noting about this study. One notable constraint is the limited sample size, consisting of only ten healthy volunteers. While this may seem insufficient for a comprehensive analysis, it is important to recognize the significant logistical challenges inherent in conducting a cross-scanner study. Coordinating scans on the same day across different MRI systems requires precise scheduling and availability, which is difficult to achieve. Despite the modest sample size, the insights gained from this initial cohort are still valuable, providing important preliminary data on the consistency of MagDensity measurements across different scanners. Future studies with larger and more diverse populations are warranted. Additionally, we have retrospectively identified that the subjects recruited for this study were of high BD (all greater than 40%). This was likely because the sample of healthy volunteers consisted mostly of young medical professionals, as the recruitment flyer was posted in the local medical school and hospital. Future studies will aim to recruit subjects with lower expected BD (e.g., post-menopause, higher weight). Furthermore, while we included three widely used clinical MRI systems, our study was not designed to isolate the individual effects of vendor, field strength, or imaging protocol on BD measurements. The two Siemens scanners were similar, while the GE system differed in all three dimensions. As a result, it remains unclear how much each factor individually contributed to the observed measurement differences. That said, this configuration allowed us to explore performance under both similar and more variable imaging conditions, including a scenario where multiple technical factors varied simultaneously, which is common in real-world multi-center studies. Ideally, we would have liked to include more combinations, such as scanners from different vendors with similar settings or same-vendor scanners at different field strengths. However, due to the need to schedule all scans within the same day for each subject, adding more scanners was not feasible. Despite these constraints, the bias introduced by cross-platform variation was small and could be effectively corrected through linear calibration, reducing inter-scanner differences to within ±0.2%. While further studies with a broader range of scanner configurations are warranted, these findings support the feasibility of achieving consistent BD measurements with MagDensity across different platforms. In future applications, more scalable strategies, such as reference subject scans, site-specific correction factors, or phantom-based standardization, may provide practical alternatives to support harmonization across platforms without requiring repeated imaging of individual patients.

## Conclusion

In conclusion, the quantitative measure of MRI-based BD—MagDensity—exhibited high robustness within the same field strength and vendor (different models) and demonstrated promising reliability following leave-one-out calibration across different vendors, scanner models, and field strengths. Rather than asserting full standardization, our findings provide proof-of-concept that scanner-specific variability can be effectively mitigated through calibration. This technique represents a meaningful step toward improving the consistency and reliability of MR-based BD measurements across platforms, which could facilitate broader clinical adoption. Future studies could aim to validate these findings in a larger and more diverse cohort and across a broader array of MRI platforms.

## Supporting information

S1 Table Supporting information. (XLSX)
